# Supplementary material for: Rare phenomena of central rhythm and pattern generation in a case of complete spinal cord injury
Source: Nat Commun. 2023 Jun 6;14:3276. doi: 10.1038/s41467-023-39034-y (PMC10244420; doi:10.1038/s41467-023-39034-y)
Supplement: Supplementary file 1 — Supplementary Information [file 41467_2023_39034_MOESM1_ESM.pdf]

Rare phenomena of central rhythm and pattern generation in a case of complete spinal cord injury

Supplementary Information

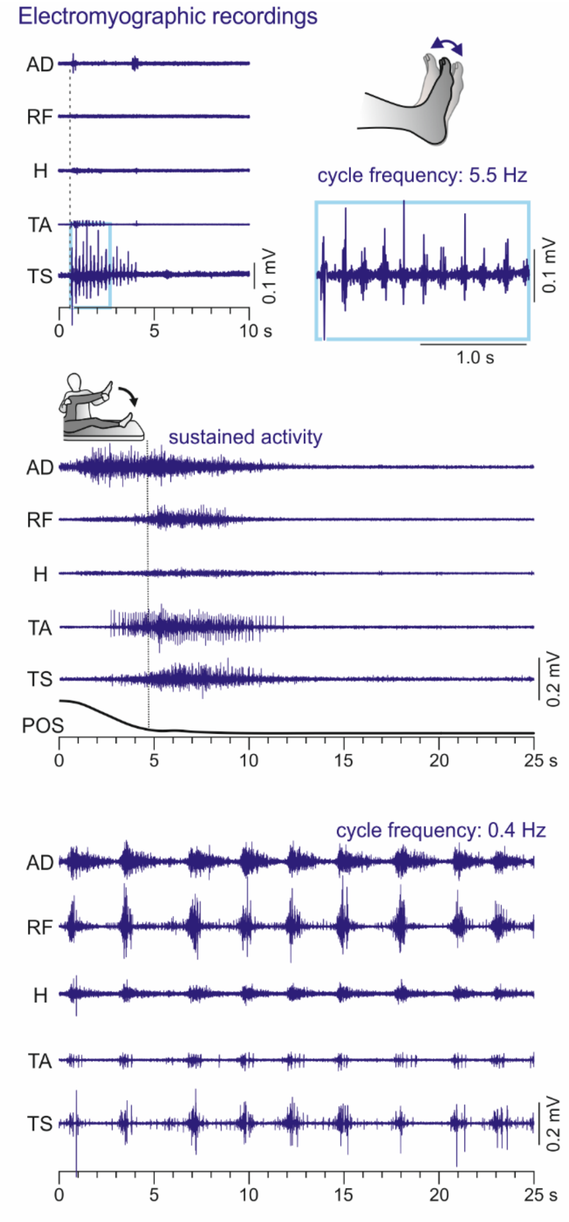

Achilles clonus

a series of brief contractions of the calf muscle caused by a brisk dorsiflexion of the foot. It appears as an involuntary oscillatory motion of the foot around the ankle with a cycle frequency **between 5 Hz and 8 Hz** and typically lasts for a few seconds.

The prevailing theory is a self-perpetuating reactivation of the stretch reflex pathway, which has become hyperactive following an upper motoneuron lesion, with each beat causing the next.

Muscle spasm

a continuous muscle activation caused by passive joint movements or stimulation of the skin. It appears as sustained contractions of multiple muscles that outlast the input that triggered them by many seconds, but **without recurring rhythmic behavior**.

It involves an increased expression of intrinsically sustained depolarizations (plateau potentials) and self-sustained firing of motoneurons. Suggested interneurons that coordinate the multi-muscle activation patterns may include a specific sub-population of pattern-coordinating CPG neurons. However, these interneurons do not themselves intrinsically oscillate or initiate rhythmic behaviour.

Bussel-Calancie type of **spinal myoclonus** in complete SCI

a sensory-evoked or spontaneously emerging rhythmic multi-muscle activity with a cycle frequency **between 0.3 Hz and 0.6 Hz**, reported in the literature in two individuals with motor-incomplete SCI* and six with complete SCI. In complete SCI, it largely involved synchronous discharges across muscles. This resulted in slow, involuntary rhythmic co-contractions that stiffened the joints, without appreciable movements other than slight internal rotation of the hips. The activity did not resemble stepping.

An involvement of the CPG has been proposed. Our data suggest a major contribution of the spinal circuitry underlying the generation of muscle spasms.

Supplementary Fig. 1. Definitions of Achilles clonus, muscle spasm, and spinal myoclonus. *In the two individuals with motor-incomplete SCI, the rhythmic activity alternated the between left and right sides and resulted in involuntary stepping-like movements in the supine position. Such a pattern of spinal myoclonus suggests a higher-level manifestation of the spinal circuits underlying the rhythmic activity

compared to the circuits generating spinal myoclonus in individuals with complete SCI, including the subject of the present study. AD, adductors; CPG, central pattern generator; H, hamstrings muscle group; POS, knee position; RF, rectus femoris; SCI, spinal cord injury; TA, tibialis anterior; TS, triceps surae muscle group.

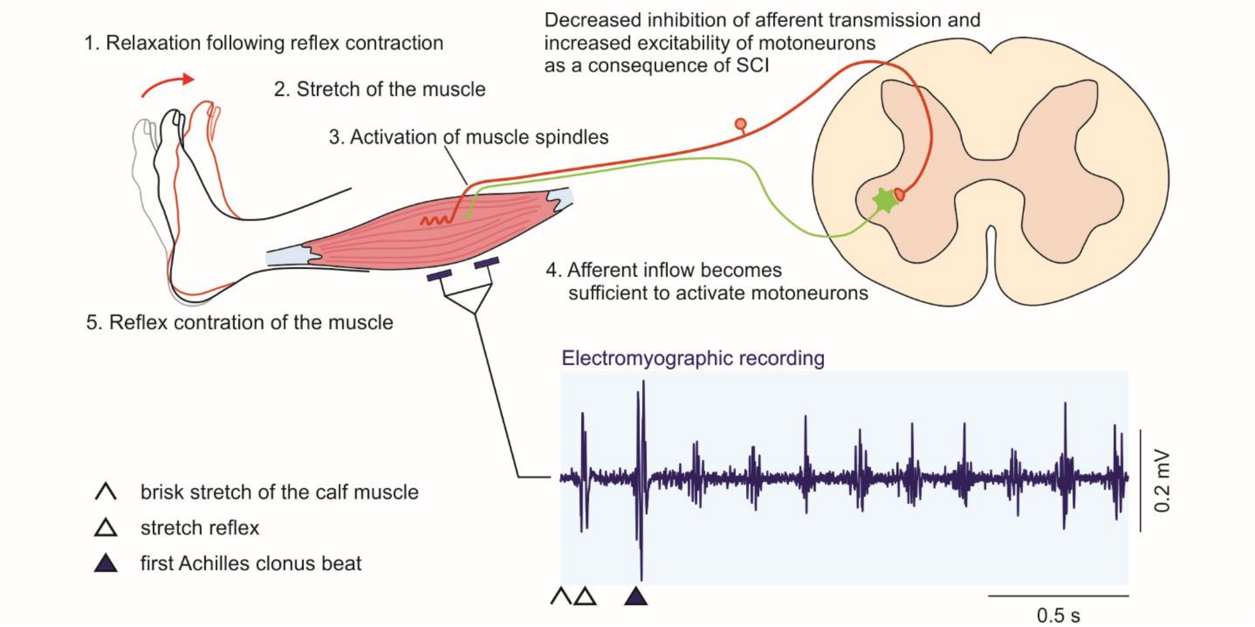

Supplementary Fig. 2. Achilles clonus and its underlying mechanisms. Achilles clonus is one of the clinically observed components of spinal spasticity in chronic spinal cord injury (SCI). It is defined as involuntary, oscillatory muscle contractions at 5–8 Hz that occur following a muscle stretch in people with lesions of the descending motor tracts[1,2]. Achilles clonus is typically accompanied by other signs of spasticity, including increased muscle tone and tendon jerks, reflecting stretch reflex hyper-excitability[3]. The prevailing theory of the underlying mechanism is a self-sustaining oscillation generated by recurrent activation of the stretch reflex. According to this theory, muscle lengthening immediately following a brief contraction (i.e., when the muscle relaxes) results in reafferent activation of muscle spindles. Due to a pathologically increased excitability of the ankle stretch reflex pathway, this proprioceptive feedback input is sufficient to reach motoneuron firing threshold, resulting in a self-excitation of the closed feedback loop[3]. The theory of this peripheral reflex rather than a central mechanism is supported by a number of observations. The latency from muscle stretch to the onset of subsequent clonus-related electromyographic activity is consistent with the conduction time in the stretch reflex pathway[3]. Lower clonus frequencies are observed in individuals with longer legs and hence longer reflex pathways[2]. The magnitude of the electromyographic activity of a clonus beat influences the onset and magnitude of the subsequent beat[2]. The magnitude of an externally applied load that causes ankle dorsiflexion correlates with the oscillation frequency of the induced clonus[1,3]. Partial block of large-diameter afferent fibers from the calf muscles by compression of the leg with a cuff, without affecting conduction in the efferent motor fibers, abolishes Achilles clonus[1]. Perhaps the most important feature is that, before the Achilles clonus disappears, there is a progressive reduction in its frequency, associated with the prolonged compression and the reduction in the conduction properties of the proprioceptive fibers. This observation demonstrates that clonus frequency is not driven by central oscillators. Importantly, although activity in muscles other than the calf muscles may be present during an Achilles clonus, they are not necessary to maintain the clonus[2,3].

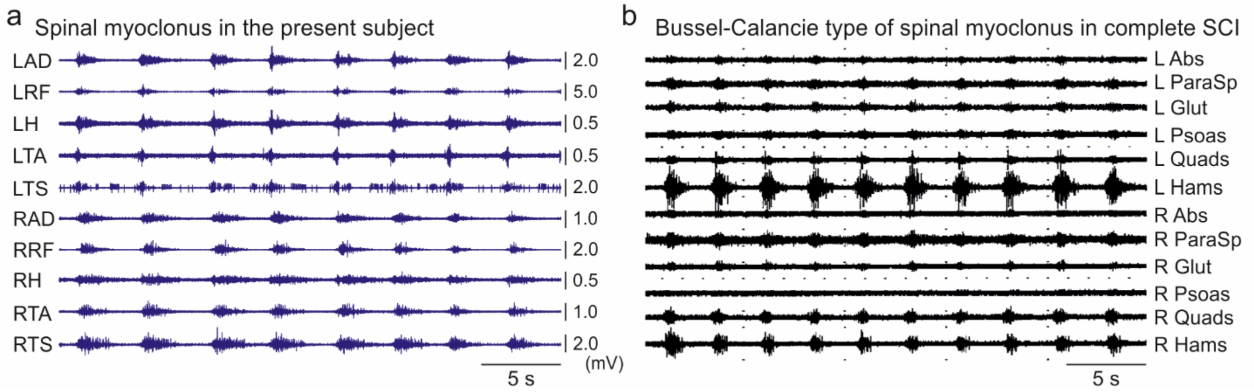

Supplementary Fig. 3. A rare type of spinal myoclonus. Spinal myoclonus is a form of involuntary muscle activity that has been broadly categorized as spinal segmental[4] or propriospinal myoclonus[5]. Spinal segmental myoclonus is a semi-rhythmic or rhythmic activity of muscle groups innervated by one or few contiguous spinal cord segments, predominantly affecting either the upper or lower limbs. The activation of the muscles is synchronous with a range of rhythm-cycle frequencies of 1–3 Hz[4,6]. The abnormal oscillations are thought to result directly from lesions or pathophysiological changes within the segments that innervate the affected muscles[6,7]. The definition of spinal segmental myoclonus excludes rhythmic activity following a release phenomenon (from brain control) of spinal generators caudal to a spinal cord lesion[6]. Propriospinal myoclonus involves flexion of the neck and trunk, and less commonly, proximal limb muscles. It is thought to arise from a spinal generator located in one or a few adjacent thoracic spinal cord segments and to spread from segment to segment, sequentially involving additional muscles with increasing delays[5]. Propriospinal myoclonus occurs as single jerks or irregularly every 20 s to a few minutes, whereby each jerk can be polymyoclonic, i.e., their electromyographic activity is composed of repetitive short beats at frequencies of 1–7 Hz. It has been proposed that propriospinal myoclonus may result from partial release of a thoracic pattern generator involving rostral and caudal spinal cord segments through long propriospinal pathways[8]. Neither of these types of spinal myoclonus can adequately account for the self-sustained rhythmic electromyographic activity in the present study. Rather, our examples (**a**) closely resemble the Bussel-Calancie type of rhythmic spinal myoclonus (**b**) described in six individuals with chronic, clinically complete SCI, in terms of rhythm-cycle frequencies, patterns of associated electromyographic activity, and the additional presence of hip pathology[9–11]. It is noteworthy that neither the Bussel-Calancie type of spinal myoclonus in complete SCI nor the spinal myoclonus described here were locomotor-like, as they lacked alternating activity between left and right or flexors and extensors. a, Spinal myoclonus following a single cycle of passive hip and knee flexion and extension; AD, adductors; H, hamstrings muscle group; L, left; R, right; RF, rectus femoris; TA, tibialis anterior; TS, triceps surae muscle group. b, Spontaneous activity with the subject lying supine with hips extended; SCI, spinal cord injury; Abs, abdominal; Glut, gluteal; Hams, hamstrings; ParaSp, paraspinal; Quads, quadriceps. Illustration in (b) from Spinal myoclonus after spinal cord injury, Calancie B, Journal of Spinal Cord Medicine 29, 413–24, 2006[10], Taylor & Francis Ltd., reprinted by permission of the publisher (Taylor & Francis Ltd., https://www.tandfonline.com).

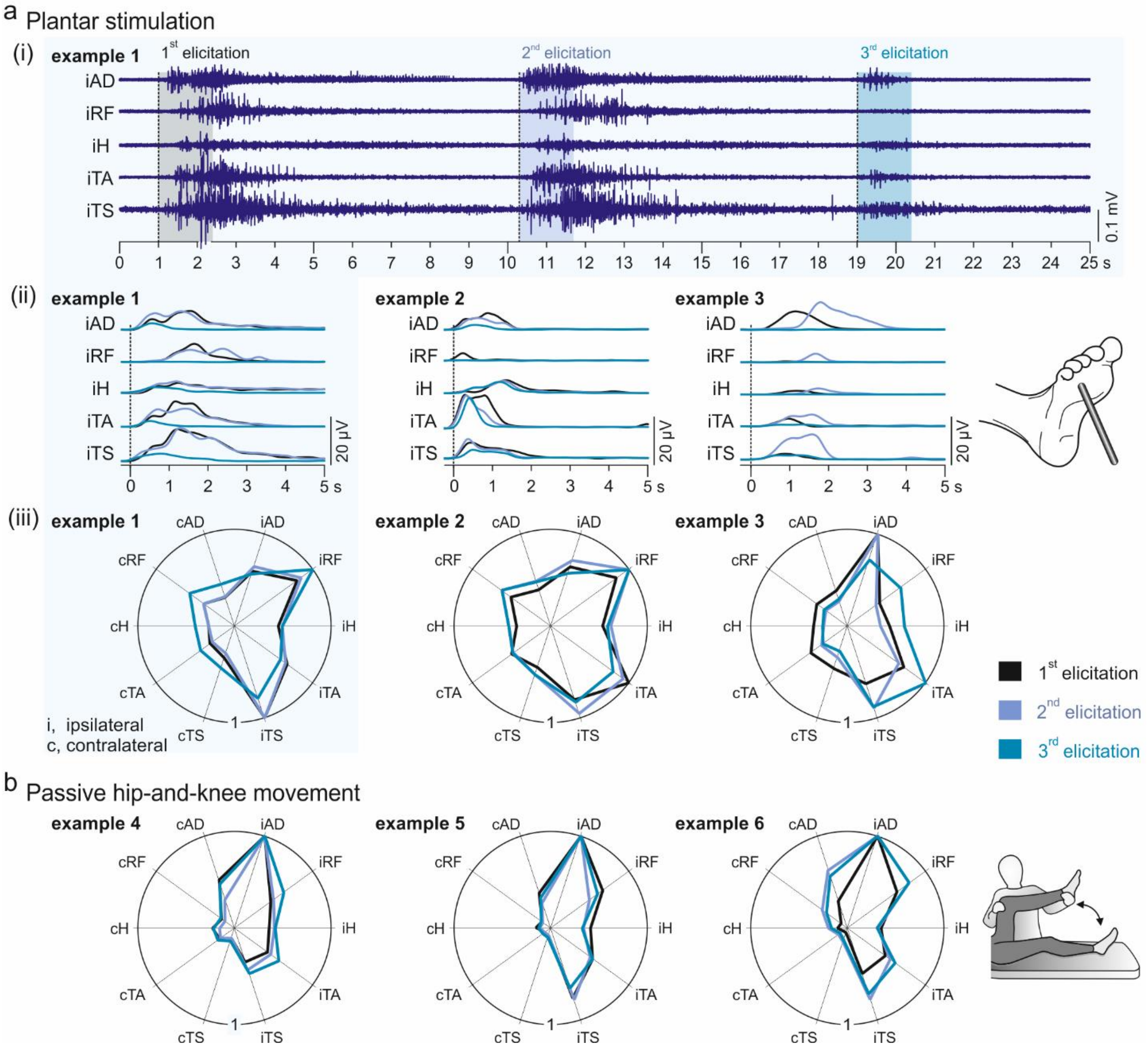

Supplementary Fig. 4. Repeatedly evoked muscle spasms have consistent multi-muscle activation patterns. When muscle spasms are triggered in close succession, they may decrease in amplitude, but their multi-muscle activation patterns remain consistent[12]. **a** (i) Plantar stimulation of the foot sole evoked spasms in the lower-limb muscles of the participant in the present study. Three consecutive repetitions are shown with electromyographic (EMG) recordings displayed for the ipsilateral AD, adductors; RF, rectus femoris; H, hamstrings muscle group; TA, tibialis anterior; and TS, triceps surae muscle group. Backgrounds indicate times of sensory stimulation. (ii) Superimposed, filtered EMG envelopes of the three repetitions of the same as well as two additional examples show the consistency of the temporal recruitment of the different muscles into the spasms. (iii) Polar plots are muscle activation patterns of re-elicited muscle spasms of the three examples in (ii). Radial axes are muscles and polar coordinates are integrated EMG activities normalized to the respective maximum per repetition. **b** Muscle activation patterns of spasms induced by imposed flexion-extension movements. Source data are provided in the Source Data file.

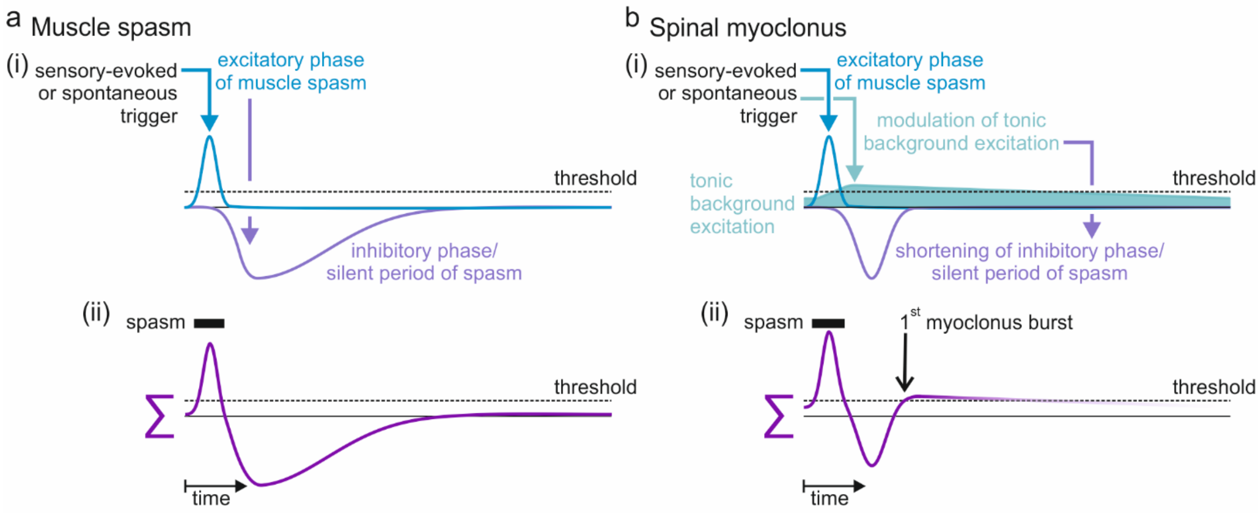

Supplementary Fig. 5. Sketches of mechanisms that may explain spinal myoclonus as repetitive muscle spasms. a (i) A sensory trigger or spontaneous alteration in spinal excitability generates excitatory neural activity that leads to a muscle spasm, followed by a prolonged period of inhibition that curtails the muscle spasm and reduces the likelihood of another identical spasm occurring in close succession[13]. (ii) Net sum of excitatory and inhibitory neural activity. **b** (i) In this case, a hip pathology or other (possibly nociceptive) signals cause additional tonic background excitation of the lumbar spinal cord circuits[10,11]. The trigger that causes the muscle spasm also modulates this ongoing excitation (e.g., indirectly through increased load or movement in the affected hip joint; assumption). The background excitation also shortens the inhibition that normally delays the onset of subsequent activity (cf., Fig. 5 of this paper and Figure 3 - figure supplement 1 in[13]). (ii) The net sum of these influences can result in a first myoclonus burst after a short time period, as soon as the background excitation overwhelms the decreasing inhibition, exceeding the threshold of the excitatory phase of a muscle spasm.

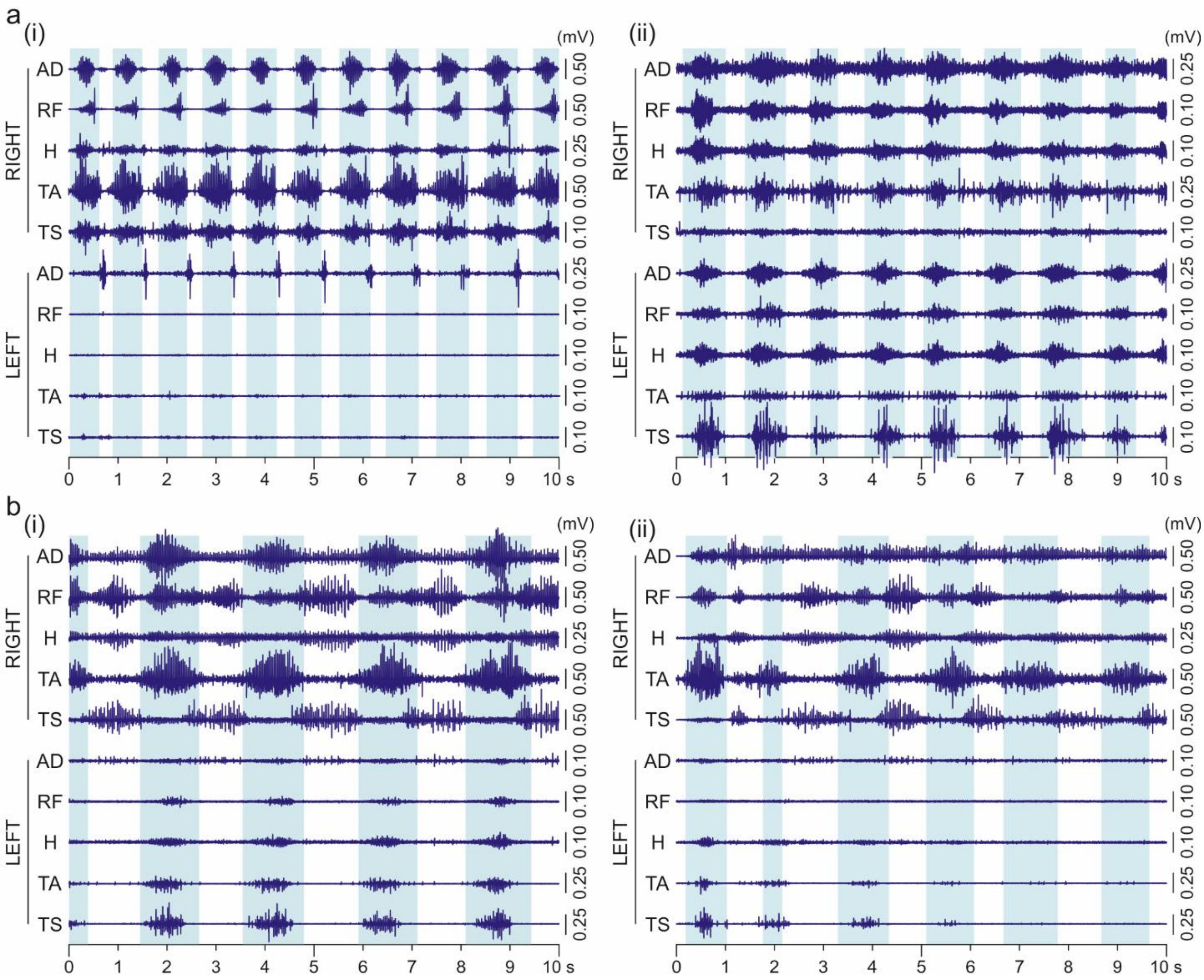

Supplementary Fig. 6. Rhythmic multi-muscle activation patterns induced by tonic epidural electrical stimulation of the lumbar spinal cord. a Fast rhythmic activity with synchronous output across muscles with cycle frequencies of (i) 0.78 Hz and (ii) 0.92 Hz. **b** More complex patterns demonstrating reciprocal relationships between extensor and flexor muscles of the right lower limb and between some of the homologous muscles of the right and left sides with cycle frequencies of (i) 0.45 Hz and (ii) 0.58 Hz. Backgrounds mark flexion-like phases of the right lower limb identified by on- and offsets of right tibialis anterior (TA) bursts. Epidural electrical stimulation (EES) was applied with unchanged parameters throughout each of the time windows displayed. a. (i) Recording EES 4, active contacts: 0+1-; stimulation frequency: 29.4 Hz, stimulation amplitude: 7 V, pulse width: 210 µs; (ii) recording EES 2, 0-3+, 90.9 Hz, 8.5 V, 210 µs; b. Recording EES 3, 0-3+, 29.4 Hz, (i) 6 V, (ii) 5 V, 210 µs. Same position of the epidural lead, with the four in-line electrodes, labelled 0 to 3 from rostral to caudal, located at the T12 vertebral level. AD, adductors; H, hamstrings muscle group; RF, rectus femoris; TS, triceps surae muscle group.

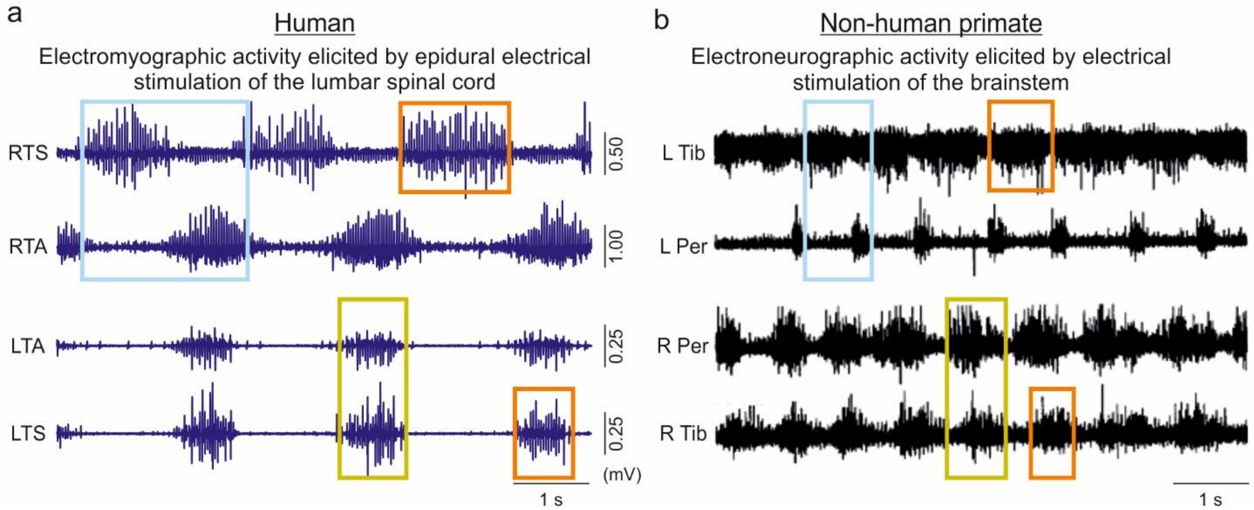

Supplementary Fig. 7. Locomotor-like electromyographic activities resemble the component fictive patterns found in the marmoset monkey. a Electromyographic activity recorded from the right (R) and left (L) tibialis anterior (TA) and triceps surae muscle group (TS) from the participant in the present study, induced by epidural electrical stimulation (EES) of the lumbar spinal cord; recording EES 3, active contacts: 0-3+, stimulation frequency: 29.4 Hz, stimulation amplitude: 6 V, pulse width: 210 µs. **b** Electroneurographic activity recorded from the left and right common tibial nerve (Tib) and common peroneal nerve (Per) of an immobilized, decerebrated marmoset monkey, induced by electrical stimulation of the brainstem; stimulation frequency: 20 Hz; stimulation amplitude: 100 µA[14]. Both examples exhibit reciprocal activity in antagonistic muscles or their supplying nerves of one side (blue rectangles) as well as in a pair of homologous muscles or their supplying nerves of the left and right side (orange rectangles). However, the full locomotor pattern was not achieved in either case as reflected by the in-phase activity in some antagonistic muscles or their supplying nerves (yellow rectangles). Illustration in (b) from Pharmacologically evoked fictive motor patterns in the acutely spinalized marmoset monkey (Callithrix jacchus), Fedirchuk B, Nielsen J, Petersen N, Hultborn H, Experimental Brain Research 122, 351–61, Springer Nature, 1998[14], reproduced with permission from SNCSC.

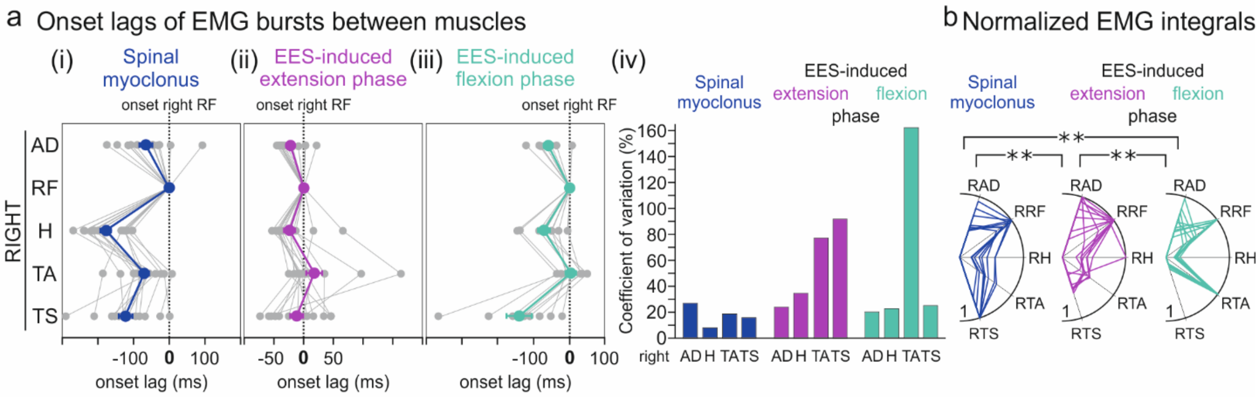

Supplementary Fig. 8. Comparison of muscle recruitment patterns during episodes of spinal myoclonus and extension- and flexion-like phases of epidural stimulation-induced rhythmic activities. a Relative onset lags of electromyographic (EMG) bursts aligned with respect to the onset of EMG bursts in the right rectus femoris (RF), shown for (i) n =11 spinal myoclonus examples and (ii) n =11 extension- and (iii) n =10 flexion-like phases of epidural electrical stimulation (EES)-induced rhythmic EMG activity. Grey lines are individual spinal myoclonus and EES examples; blue, magenta, and green lines are the respective mean values (± SE). For spinal myoclonus, there was no significant interaction between example and muscle, ANOVA, $F(38;1) = 21.151$, $p = 0.171$, $\eta_p^2 = 0.999$. For the EES examples, this interaction was significant for the extension-like phases, $F(37;1) = 5.807$, $p < .001$, $\eta_p^2 = 0.995$, but not for the flexion-like phases, $F(31;1) = 0.050$, $p = 1.000$, $\eta_p^2 = 0.608$. The sequence of muscle recruitment differed between spinal myoclonus and the extension-like phases, $F(3;103) = 6.240$, $p < .001$, $\eta_p^2 = 0.154$ (compare (i) and (ii)), and the flexion-like phases of EES-induced rhythmic activity, $F(3;84) = 4.333$, $p = .007$, $\eta_p^2 = 0.134$ (compare (i) and (iii)). The sequence of muscle recruitment also differed between the extension- and flexion-like phases of the EES examples, $F(3;83) = 5.877$, $p = .001$, $\eta_p^2 = 0.175$ (compare (ii) and (iii)). (iv) Variability of onset lags per muscle across examples, expressed by the respective coefficients of variation. **b** Polar plots show muscle activation patterns of spinal myoclonus and extension- and flexion-like phases of EES-induced rhythmic activities. Radial axes are muscles and polar coordinates are integrated EMG activities normalized to the respective maximum per example. There was no significant interaction between example and muscle for spinal myoclonus, $F(39;1) = 0.497$, $p = .195$, $\eta_p^2 = 0.951$, nor for the extension-like phases, $F(39;1) = 16.174$, $p = .195$, $\eta_p^2 = 0.998$, or the flexion-like phases of EES-induced rhythmic EMG activities, $F(35;1) = 57.208$, $p = .104$, $\eta_p^2 = 0.999$. However, the pattern of muscle recruitment differed significantly between spinal myoclonus and EES examples; compared to extension-like phases, $F(4;95) = 23.269$, $p < .001$, $\eta_p^2 = 0.495$; and to flexion-like phases, $F(4;90) = 51.537$, $p < .001$, $\eta_p^2 = 0.696$. The pattern of muscle recruitment also differed significantly between extension- and flexion-like phases of the EES examples, $F(4;95) = 21.231$, $p < .001$, $\eta_p^2 = 0.472$. AD, adductors; H, hamstrings muscle group; R, right; RF; rectus femoris; TA, tibialis anterior; TS, triceps surae muscle group; **, p < .001. All statistical tests were two-sided. Post-hoc tests were Bonferroni-corrected to adjust for multiple comparisons. Source data are provided in the Source Data file.

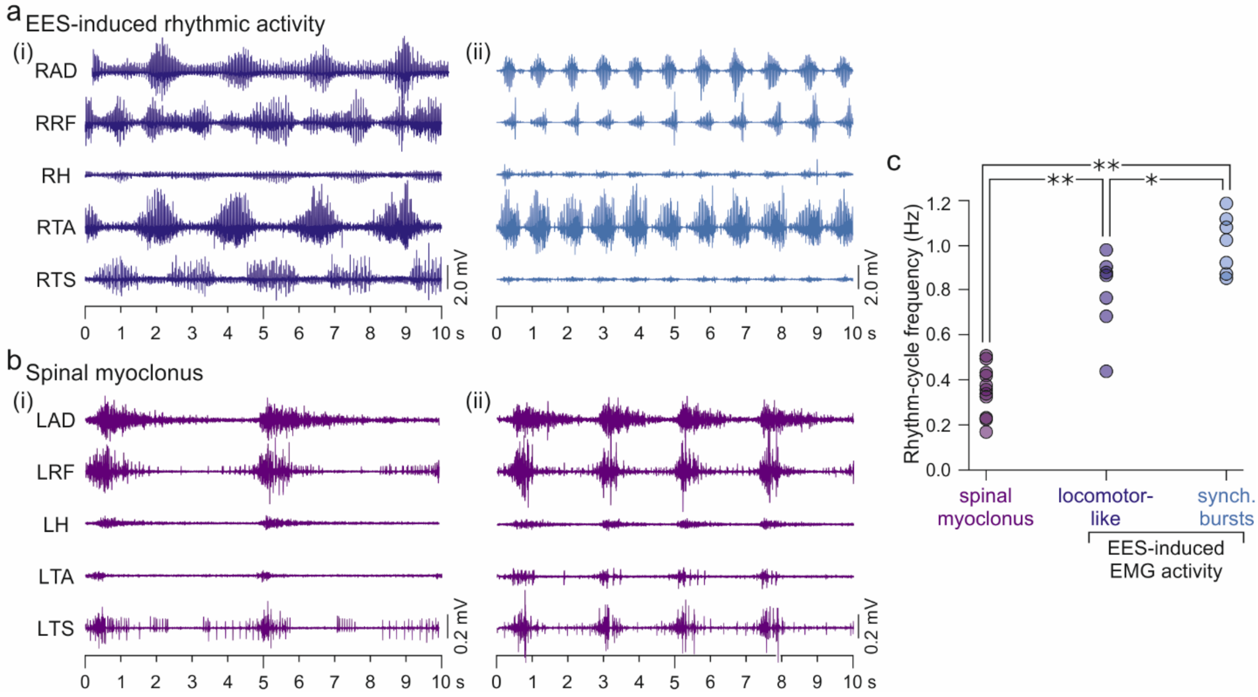

Supplementary Fig. 9. Epidural stimulation-induced motor patterns change with increasing cycle frequency in contrast to the consistent spinal myoclonus patterns. a Examples of electrical epidural stimulation (EES)-induced rhythmic activity with (i) a slow rhythm-cycle frequency and locomotor-like pattern and (ii) a fast rhythm-cycle frequency and synchronous bursts of activity across muscles, occurring in-phase with the dominant activity in the flexor muscle tibialis anterior (TA). a(i), recording EES 3, active contacts: 0-3+, stimulation frequency: 29.4 Hz, stimulation amplitude: 6 V, pulse width: 210 µs; and a(ii), recording EES 4, 0+1-, 29.4 Hz, 7 V, 210 µs. **b** Examples of slow (i) and fast (ii) spinal myoclonus; examples 2 b(i) and 9 b(ii) (cf., Supplementary Table 2). **c** Rhythm-cycle frequencies of n = 11 spinal myoclonus examples as well as of n = 7 EES-induced rhythmic activities with locomotor-like patterns and n = 7 synchronous (synch.) bursts of activity. To allow statistical comparisons, we increased the sample size of EES examples by including episodes of rhythmic activity consisting of at least four complete cycles, even if they had lasted less than 10 s. Remarkably, all but one example of EES-induced rhythmic activities had rhythm-cycle frequencies higher than those of spinal myoclonus. Notably, a linear mixed model revealed significant differences between the rhythm-cycle frequencies of spinal myoclonus, EES-induced locomotor-like activity, and EES-induced synchronous bursting, $F(2;22) = 52.988$, $p < .001$, $\eta_p^2 = 0.828$. Bonferroni-corrected post-hoc pairwise comparisons demonstrated that spinal myoclonus had lower rhythm-cycle frequencies than both patterns of EES-induced rhythmic EMG activity (**, both $p < .001$). Furthermore, rhythm-cycle frequencies of the EES-induced locomotor-like EMG activities were lower than those of the EES-induced synchronous bursting (*, $p = .021$). AD, adductors; H, hamstrings muscle group; L, left; R, right; RF; rectus femoris; TS, triceps surae muscle group. Source data are provided in the Source Data file.

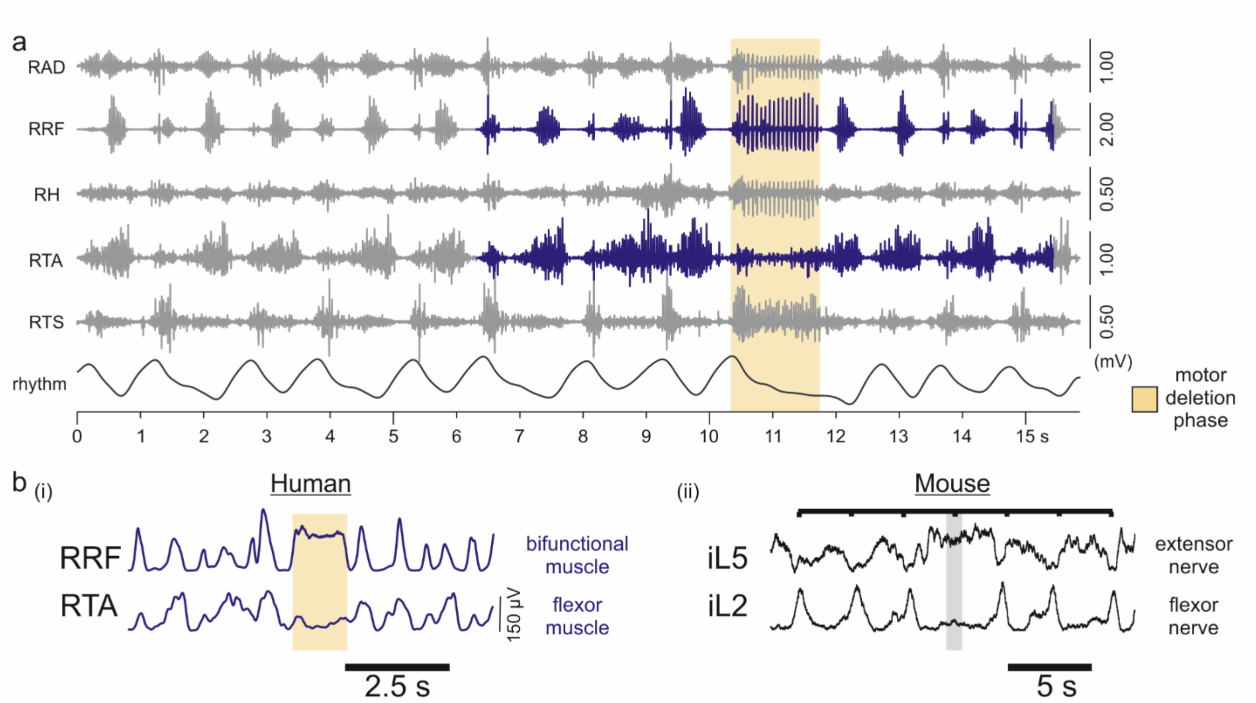

Supplementary Fig. 10. Motor deletions in tibialis anterior resemble flexor deletions in the isolated mouse spinal cord. a Electromyographic (EMG) activity of the right (R) adductor (AD), rectus femoris (RF), hamstrings muscle group (H), tibialis anterior (TA), and triceps surae muscle group (TS) induced by epidural electrical stimulation (EES) of the lumbar spinal cord of the participant in the present study; same example as Fig. 7a(ii); recording: EES 4, active contacts: 0+1-, stimulation frequency: 29.4 Hz, stimulation amplitude: 8 V, pulse width: 210 µs; rhythm, inclinometer recording from the knee. The background marks a time window containing a motor deletion in TA, accompanied by tonic activity in the other muscles. Sections of EMG activity in blue are shown as filtered EMG envelopes in b(i). **b**(i) The filtered EMG envelopes of the flexor muscle TA and the bifunctional RF resemble flexor deletions in the mouse in a remarkable way[15]. (ii) Spontaneous flexor deletion during NMDA/5-HT-induced fictive locomotion in the isolated neonatal mouse spinal cord[15]. The traces are smoothed and rectified motoneuron activity recorded extracellularly from ipsilateral lumbar L2 and L5 ventral roots. L2 recordings show predominantly flexor motoneuron activity, while L5 recordings show predominantly extensor activity. The bars above the traces indicate the expected timing of the bursts if the rhythm were unperturbed during the deletion. The recordings show a non-resetting flexor deletion (indicated by the light grey bar) that is accompanied by tonic activity in the ipsilateral extensor root. Illustration in (b)(ii) from Neuronal activity in the isolated mouse spinal cord during spontaneous deletions in fictive locomotion: insights into locomotor central pattern generator organization, Zhong G, Shevtsova NA, Rybak IA, Harris-Warrick RM, Journal of Physiology 590, 4735–59, 2012[15], John Wiley and Sons, reprinted by permission of the publisher (https://physoc.onlinelibrary.wiley.com), © 2012 The Authors. The Journal of Physiology © 2012 The Physiological Society.

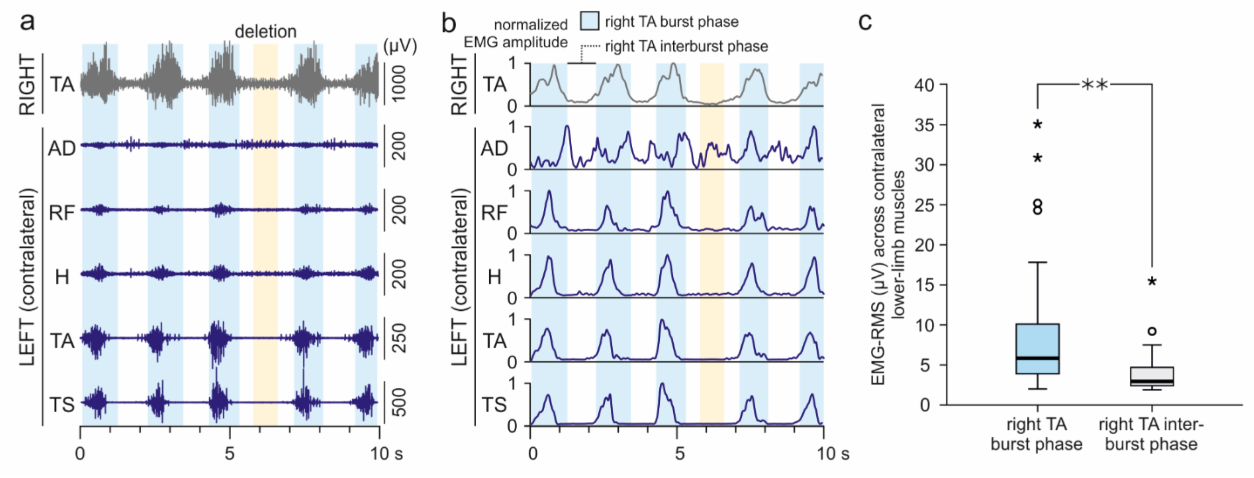

Supplementary Fig. 11. Electromyographic bursts of epidural stimulation-induced activity in contralateral lower-limb muscles were in-phase with the flexor activity in the right lower limb. a Exemplary electromyographic (EMG) recordings from left lower-limb muscles shown aligned with right tibialis anterior (TA) activity. The blue backgrounds mark the right TA burst phases, the yellow background is the deletion phase. **b** Filtered EMG envelopes, normalized to the respective maximum per muscle, indicate that the activity across the contralateral lower-limb muscles was largely synchronized with the bursts of the right TA. Same example shown as in a, recording EES 3, active contacts: 0-3+, stimulation frequency: 29.4 Hz, stimulation amplitude: 6V, pulse width: 210 µs. AD, adductors; H, hamstrings muscle group; RF, rectus femoris; TS, triceps surae muscle group. **c** EMG-root mean square (RMS) values across the contralateral lower-limb muscles during the burst and interburst phases of the regular rhythmic activity in the right TA, illustrated by box plots. Bold horizontal lines within boxes are medians, boxes span the inter-quartile range (IQR). Whiskers extend to the lowest and largest values that are not outliers (values 1.5–3 times the IQR; circles) or extreme values (values > 3 times the IQR; asterisks). Statistical analysis, considering all muscles and six available examples with bilateral rhythmic activity, confirmed significantly greater EMG activities across contralateral muscles during right TA burst than interburst phases, two-sided Wilcoxon test, $z = -4.351$, $p < .001$, $r = 0.794$. **, $p < .001$. EES, epidural electrical stimulation. Source data are provided in the Source Data file.

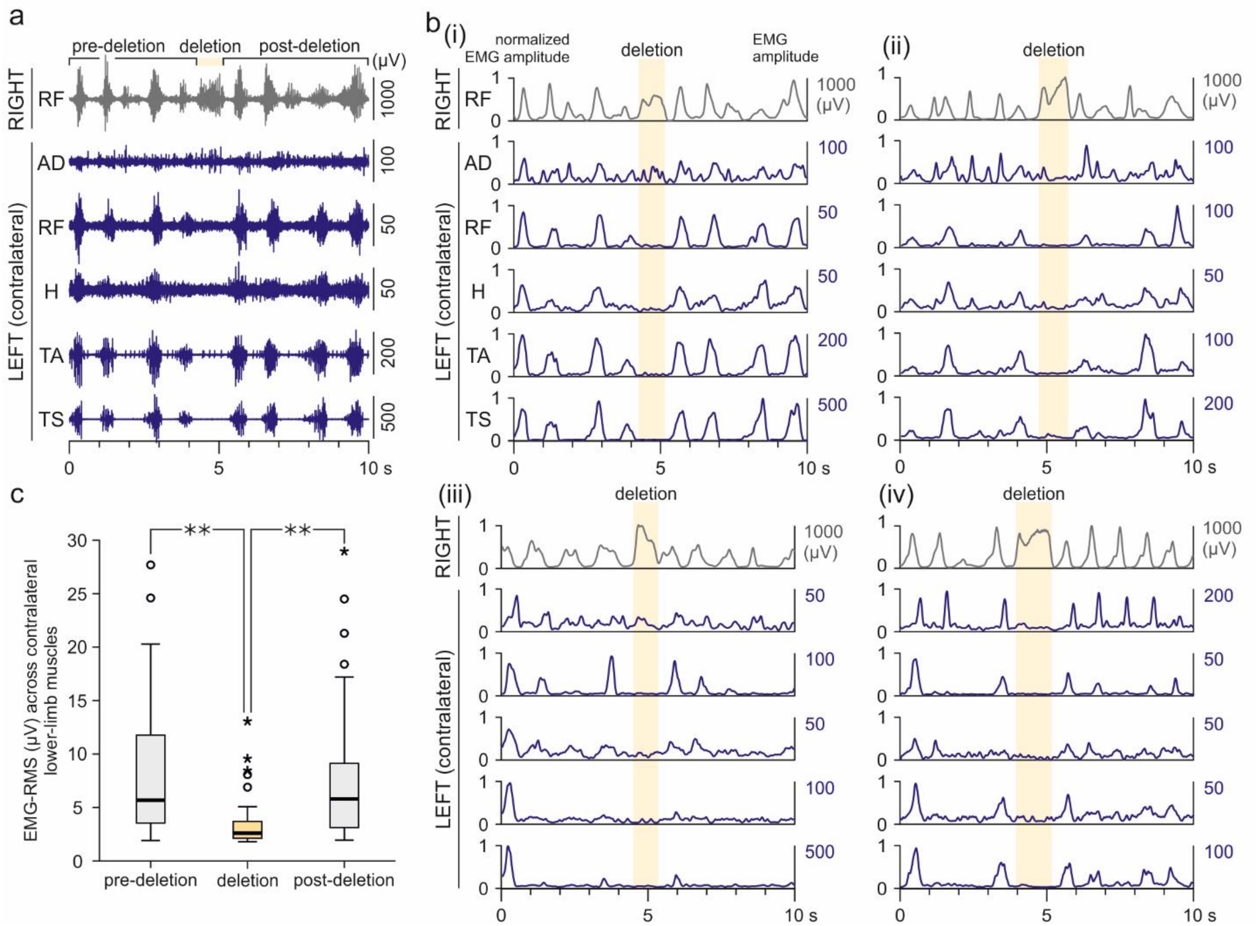

Supplementary Fig. 12. Impact of unilateral flexor deletions on epidural stimulation-induced activity in the contralateral lower-limb muscles. a Exemplary electromyographic (EMG) recordings from the left lower-limb muscles shown aligned with the activity of the right rectus femoris (RF) used to mark the phase of the motor deletion in the tibialis anterior (TA). **b** Filtered EMG envelopes normalized to the respective maximum per muscle derived from four examples indicate markedly reduced activity across contralateral lower-limb muscles during flexor deletions (backgrounds). Scaling on the right side of each trace shows that the EMG activity on the contralateral side was considerably lower in amplitude compared to the right RF. a and b(i), recording EES 3, active contacts: 0-3+, stimulation frequency: 29.4 Hz, stimulation amplitude: 7V, pulse width: 210 µs; b(ii)–(iv), recording EES 4, active contacts: 0+1-, stimulation frequency: 29.4 Hz, stimulation amplitude: 10 V, 9 V, 8 V, respectively, pulse width: 210 µs. AD, adductors; H, hamstrings muscle group; TS, triceps surae muscle group. **c** EMG-root mean square (RMS) values of the pre-deletion, deletion, and post-deletion phases, illustrated by box plots. Bold horizontal lines within boxes are medians, boxes span the inter-quartile range (IQR). Whiskers extend to the lowest and largest values that are not outliers (values 1.5–3 times the IQR; circles) or extreme values (values > 3 times the IQR; asterisks). Statistical analysis, considering all muscles and six available examples with bilateral rhythmic activity, confirmed significantly reduced EMG activities across contralateral muscles during phases of unilateral flexor deletions compared to pre- and post-deletion phases, two-sided Friedman test, $\chi^2(30) = 21.227$, $p < .001$, $W = 0.354$. Bonferroni-corrected post-hoc pairwise comparisons revealed significant differences between deletion phases and both pre- and post-deletion (both $p < .001$), but not between pre- and post-

deletion ($p = 1.000$). **, $p < .001$. EES, epidural electrical stimulation. Source data are provided in the Source Data file.

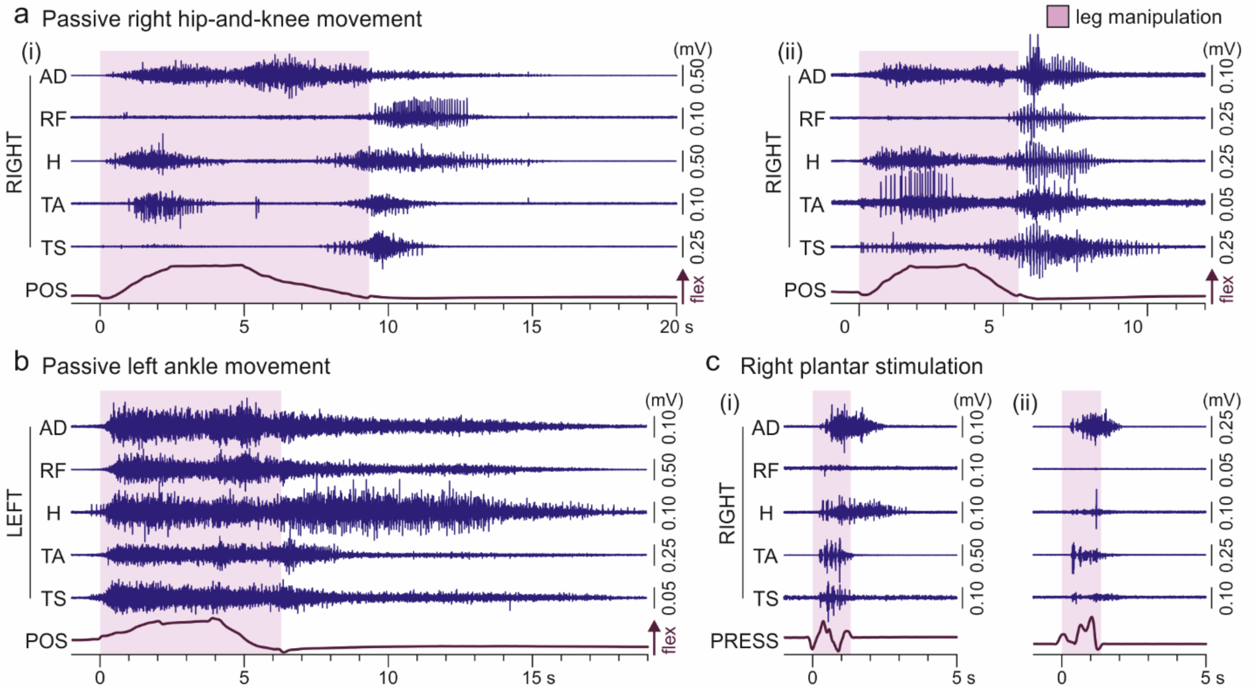

Supplementary Fig. 13. Muscle spasms induced in participants with chronic motor-complete spinal cord injury in a previous study never evolved into spinal myoclonus[16]. Muscle spasms induced in four individuals **a** by imposed flexion-extension movement of the right lower limb; POS, knee position; **b** by passive flexion-extension movement of the left ankle; POS, foot position; and **c** by stroking the right plantar surface with a blunt rod in a fashion to test the Babinski reflex; PRESS, pressure transducer signal. Recordings derived from a. (i), participant 4; (ii) participant 6; b. participant 5; and c. (i), participant 3; (ii) participant 4 in Danner et al., 2015[16]. AD, adductors; flexion, flexion; H, hamstrings muscle group; RF, rectus femoris; TA, tibialis anterior; TS, triceps surae muscle group.

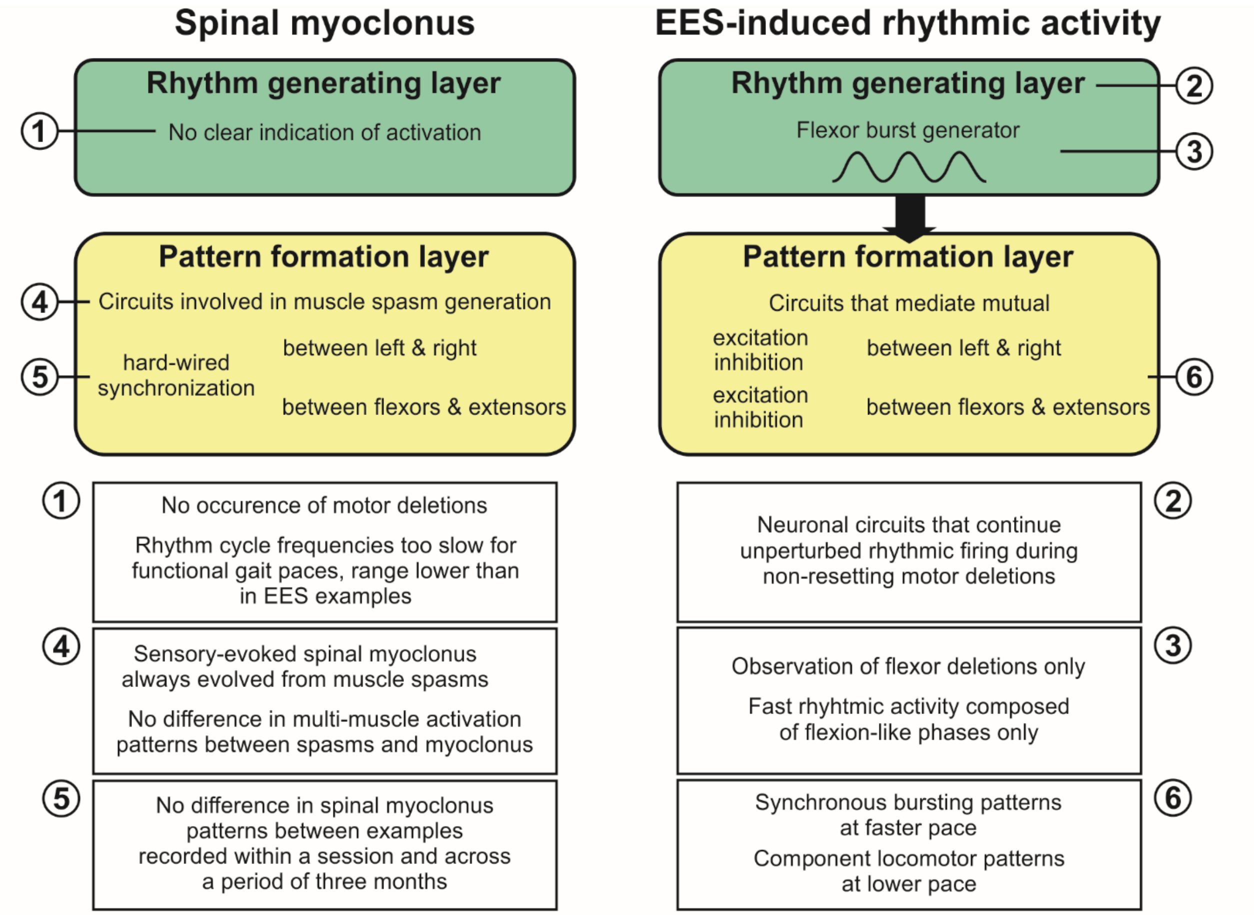

Supplementary Fig. 14. Spinal myoclonus and epidural stimulation-induced rhythmic activity are generated by different elements of the lumbar spinal motor circuits. Summary of hypothetical mechanisms suggested by the data of the present study. Spinal myoclonus showed similarities to muscle spasms but essential differences from epidural electrical stimulation (EES)-induced activities, both in rhythm and pattern. The data did not provide clear indication for the activity of intrinsically rhythm-generating elements in spinal myoclonus. The spinal myoclonus patterns did not yield left-right or flexor-extensor alternations. At the same time, the rhythmic multi-muscle patterns induced by EES hinted at a flexor-biased activation of rhythm-generating circuits, with distribution of activity to different muscles through a downstream pattern formation layer. Data supporting these assumptions are listed in the corresponding numbered boxes. Furthermore, the re-evaluation of data from a previous study[16] showed that there was no interdependence between the occurrence of spinal myoclonus and rhythmic activity induced by EES .

Supplementary Table 1. Examination according to the international standards for neurological classification of spinal cord injury.

**Upper limb motor scores**		
	Right	*Left*
C5	5	4
C6	5	4
C7	5	4
C8	5	4
T1	5	4
**Lower limb motor scores**		
	Right	*Left*
L2	0	0
L3	0	0
L4	0	0
L5	0	0
S1	0	0

	**Sensory scores**			
	Light Touch		Pin Prick	
	Right	*Left*	*Right*	*Left*
C2	2	2	2	2
C3	2	2	2	2
C4	2	2	2	2
C5	2	2	2	2
C6	2	2	2	2
C7	2	2	2	2
C8	2	2	2	2
T1	2	2	2	2
T2	2	2	2	2
T3	2	2	2	2
T4	2	2	1	2
T5	2	2	1	1
T6	2	2	1	1
T7	1	1	0	0
T8	0	0	0	0
T9	0	0	0	0
T10	0	0	0	0
T11	0	0	0	0
T12	0	0	0	0
L1	0	0	0	0
L2	0	0	0	0
L3	0	0	0	0
L4	0	0	0	0
L5	0	0	0	0
S1	0	0	0	0
S2	0	0	0	0
S3	0	0	0	0
S4–5	0	0	0	0

Voluntary anal contraction: No; Deep anal pressure: No

Supplementary Table 2. Examples of self-sustained rhythmic activity in paralyzed lower-limb muscles.

Example	Neurol. exam.	Genesis	Involved lower-limb muscles	
1	1	Passive left ankle movement	Left	AD, RF, H, TA, TS
			Right	--
2	2	Passive right hip and knee movement	Left	AD, RF, H, TA, TS
			Right	AD, RF, H, TA, TS
3	3	Passive right hip and knee movement	Left	--
			Right	AD, RF, H, TA
4	4	Cutaneous input evoked spasms (right)	Left	AD, RF, H, TA, TS
			Right	AD, RF, H, TA, TS
5	4	Cutaneous input evoked spasms (right)	Left	AD, RF, H, TA, TS
			Right	AD, RF, H, TA, TS
6	4	Spontaneous	Left	AD, RF, H, TA, TS
			Right	AD, H, TA, TS
7	4	Spontaneous	Left	AD, RF, H, TA, TS
			Right	AD, RF, H, TA
8	4	Spontaneous	Left	AD, RF, H, TA, TS
			Right	AD, RF, H, TA, TS
9	4	Spontaneous	Left	AD, RF, H, TA, TS
			Right	AD, RF, H, TA, TS
10	5	Passive right hip and knee movement	Left	AD, RF, H
			Right	AD, H, TA, TS
11	6	Passive left ankle movement	Left	AD, RF, H, TA, TS
			Right	AD, RF, H, TA, TS

Neurol. exam., neurological examination (sorted in chronological order); AD, adductors, RF, rectus femoris; H, hamstrings muscle group; TA, tibialis anterior; TS, triceps surae muscle group

Supplementary Table 3. Median cycle frequency (IQR) of self-sustained rhythmic activity.

Example	Muscle (left)	Median cycle frequency (IQR), (Hz)	Muscle (right)	Median cycle frequency (IQR), (Hz)	Results of statistical comparison between muscles
1	AD	0.33 (0.32–0.40)	AD	NA	$\chi^2(4) = 1.214$ p = .876 W = .101
	RF	0.32 (0.32–0.41)	RF	NA	
	H	0.33 (0.32–0.40)	H	NA	
	TA	0.32 (0.32–0.38)	TA	NA	
	TS	0.33 (0.31–0.41)	TS	NA	
2	AD	0.24 (0.21–0.28)	AD	0.25 (0.21–0.27)	$\chi^2(9) = 3.768$ p = .926 W = .038
	RF	0.24 (0.21–0.27)	RF	0.24 (0.22–0.27)	
	H	0.24 (0.21–0.28)	H	0.25 (0.22–0.27)	
	TA	0.24 (0.21–0.28)	TA	0.24 (0.21–0.28)	
	TS	0.24 (0.21–0.28)	TS	0.24 (0.21–0.27)	
3	AD	NA	AD	0.33 (0.19–0.31)	$\chi^2(3) = 1.080$ p = .782 W = .072
	RF	NA	RF	0.35 (0.20–0.33)	
	H	NA	H	0.37 (0.19–0.37)	
	TA	NA	TA	0.34 (0.19–0.31)	
	TS	NA	TS	NA	
4	AD	0.18 (0.14–0.23)	AD	0.18 (0.14–0.22)	$\chi^2(9) = 13.230$ p = .152 W = .367
	RF	0.17 (0.14–0.23)	RF	0.18 (0.14–0.21)	
	H	0.18 (0.14–0.23)	H	0.18 (0.14–0.21)	
	TA	0.18 (0.14–0.23)	TA	0.18 (0.14–0.22)	
	TS	0.18 (0.14–0.24)	TS	0.18 (0.14–0.22)	
5	AD	0.22 (0.20–0.25)	AD	0.23 (0.20–0.24)	$\chi^2(9) = 4.246$ p = .895 W = .094
	RF	0.22 (0.20–0.25)	RF	0.22 (0.20–0.23)	
	H	0.22 (0.20–0.25)	H	0.22 (0.20–0.28)	
	TA	0.23 (0.19–0.26)	TA	0.22 (0.20–0.24)	
	TS	0.23 (0.20–0.26)	TS	0.22 (0.20–0.24)	
6	AD	0.49 (0.47–0.52)	AD	0.50 (0.47–0.53)	$\chi^2(8) = 2.805$ p = .946 W = .044
	RF	0.50 (0.47–0.53)	RF	NA	
	H	0.50 (0.48–0.53)	H	0.51 (0.47–0.52)	
	TA	0.49 (0.46–0.53)	TA	0.49 (0.45–0.53)	
	TS	0.50 (0.46–0.51)	TS	0.49 (0.46–0.54)	
7	AD	0.39 (0.31–0.46)	AD	0.39 (0.29–0.43)	$\chi^2(8) = 6.640$ p = .576 W = .277
	RF	0.39 (0.31–0.46)	RF	0.38 (0.29–0.43)	
	H	0.39 (0.30–0.46)	H	0.40 (0.38–0.43)	
	TA	0.38 (0.31–0.48)	TA	0.39 (0.30–0.44)	
	TS	0.38 (0.31–0.48)	TS	NA	
8	AD	0.49 (0.33–0.53)	AD	0.48 (0.33–0.51)	$\chi^2(9) = 7.618$ p = .573 W = .121
	RF	0.48 (0.33–0.54)	RF	0.46 (0.33–0.51)	
	H	0.48 (0.33–0.53)	H	0.48 (0.32–0.50)	
	TA	0.47 (0.33–0.52)	TA	0.47 (0.33–0.50)	
	TS	0.48 (0.33–0.53)	TS	0.47 (0.34–0.51)	
9	AD	0.42 (0.37–0.47)	AD	0.39 (0.34–0.44)	$\chi^2(9) = 4.448$ p = .879 W = .082
	RF	0.42 (0.37–0.48)	RF	0.42 (0.37–0.48)	
	H	0.42 (0.37–0.48)	H	0.42 (0.38–0.48)	
	TA	0.42 (0.37–0.48)	TA	0.42 (0.37–0.48)	
	TS	0.42 (0.37–0.48)	TS	0.40 (0.38–0.49)	
10	AD	0.42 (0.30–0.44)	AD	0.41 (0.24–0.46)	$\chi^2(6) = 8.764$ p = .187 W = .487
	RF	0.42 (0.31–0.43)	RF	NA	
	H	0.42 (0.23–0.45)	H	0.43 (0.30–0.44)	
	TA	NA	TA	0.40 (0.24–0.41)	
	TS	NA	TS	0.42 (0.31–0.43)	
11	AD	0.51 (0.44–0.51)	AD	0.51 (0.44–0.56)	$\chi^2(9) = 3.446$ p = .944 W = .128
	RF	0.51 (0.44–0.54)	RF	0.50 (0.45–0.57)	
	H	0.51 (0.45–0.55)	H	0.51 (0.44–0.53)	
	TA	0.50 (0.45–0.54)	TA	0.51 (0.44–0.51)	
	TS	0.51 (0.44–0.54)	TS	0.50 (0.45–0.52)	

NA, not applicable (no rhythmic activity or too few bursts available for statistical analysis); AD, adductors; H, hamstrings muscle group; RF, rectus femoris; TA, tibialis anterior; TS, triceps surae muscle group. Two-sided Friedman tests were used for statistical comparisons.

Supplementary Table 4. Muscle activation during flexor-biased, extensor-biased, as well as initial and later phases of motor deletions across 12 available examples of epidural electrical stimulation-induced rhythmic activities including motor deletions.

Muscle	Mean EMG integrals ± SE (mV) Flexor-biased phase	Extensor-biased phase	Deletion phase, initial division (D_1)	Deletion phase, remaining divisions (D_{2-6})	Sign. results of Bonferroni-corrected comparisons between phases
RAD	56.6 ± 2.3	37.1 ± 3.1	26.8 ± 6.7	12.0 ± 6.7	Flex. vs. all other phases, all p < .001 Ext. vs. D_{2-6}, p = .005
RRF	73.2 ± 2.3	39.4 ± 3.1	34.5 ± 6.7	40.3 ± 6.7	Flex. vs. all other phases, all p < .001
RH	14.9 ± 2.3	16.3 ± 3.1	12.4 ± 6.7	9.9 ± 6.7	none
RTA	85.6 ± 2.3	21.9 ± 3.1	11.9 ± 6.7	9.4 ± 6.7	Flex. vs. all other phases, all p < .001
RTS	12.3 ± 2.3	19.9 ± 3.1	14.5 ± 6.7	11.5 ± 6.7	none
**Sign. results of Bonferroni-corrected comparisons between muscles**	RA vs. all other muscles, all p < .001 RRF vs. RH, p < .001 RRF vs. RTS, p < .001 RRF vs. RTA, p = .002 RH vs. RTA, p < .001 RTA vs. RTS, p < .001	RA vs. RH, p < .001 RA vs. RTA, p = .006 RA vs. RTS, p = .001 RRF vs. RH, p < .001 RRF vs. RTA, p = .001 RRF vs. RTS, p < .001	none	RRF vs. RA, p = .031 RRF vs. RH, p = .015 RRF vs. RTA, p = .012 RRF vs. RTS, p = .026	

AD, adductors; Flex., flexor-biased; H, hamstrings muscle group; R, right; RF, rectus femoris; sign., significant; TA, tibialis anterior; TS, triceps surae muscle group. Mean values were compared by fitting a two-sided linear mixed model.

Supplementary references

1. Rossi, A., Mazzocchio, R. & Scarpini, C. Clonus in man: a rhythmic oscillation maintained by a reflex mechanism. *Electroencephalogr. Clin. Neurophysiol.* **75**, 56–63 (1990).
2. Wallace, D. M., Ross, B. H. & Thomas, C. K. Characteristics of Lower Extremity Clonus after Human Cervical Spinal Cord Injury. *J. Neurotrauma* **29**, 915–924 (2012).
3. Hidler, J. M. & Rymer, W. Z. A simulation study of reflex instability in spasticity: origins of clonus. *IEEE Trans. Rehabil. Eng.* **7**, 327–340 (1999).
4. Jankovic, J. Segmental Myoclonus. *Arch. Neurol.* **43**, 1025 (1986).
5. Brown, P., Thompson, P. D., Rothwell, J. C., Day, B. L. & Marsden, C. D. Axial myoclonus of propriospinal origin. *Brain* **114**, 197–214 (1991).
6. Caviness, J. N. Segmental Myoclonus. in *Hyperkinetic Movement Disorders* 221–235 (Wiley-Blackwell, 2012). doi:10.1002/9781444346183.ch15.
7. Davis, S. M., Murray, N. M., Diengdoh, J. V, Galea-Debono, A. & Kocen, R. S. Stimulus-sensitive spinal myoclonus. *J. Neurol. Neurosurg. Psychiatry* **44**, 884–888 (1981).
8. Brown, P., Rothwell, J. C., Thompson, P. D. & Marsden, C. D. Propriospinal myoclonus: Evidence for spinal 'pattern' generators in humans. *Mov. Disord.* **9**, 571–576 (1994).
9. Bussel, B. *et al.* Myoclonus in a patient with spinal cord transection. Possible involvement of the spinal stepping generator. *Brain* **111**, 1235–45 (1988).
10. Calancie, B. Spinal myoclonus after spinal cord injury. *J. Spinal Cord Med.* **29**, 413–24 (2006).
11. Nadeau, S., Jacquemin, G., Fournier, C., Lamarre, Y. & Rossignol, S. Spontaneous motor rhythms of the back and legs in a patient with a complete spinal cord transection. *Neurorehabil. Neural Repair* **24**, 377–83 (2010).
12. Sherwood, A. M., McKay, W. B. & Dimitrijević, M. R. Motor control after spinal cord injury: assessment using surface EMG. *Muscle Nerve* **19**, 966–79 (1996).
13. Bellardita, C. *et al.* Spatiotemporal correlation of spinal network dynamics underlying spasms in chronic spinalized mice. *Elife* **6**, (2017).
14. Fedirchuk, B., Nielsen, J., Petersen, N. & Hultborn, H. Pharmacologically evoked fictive motor patterns in the acutely spinalized marmoset monkey (Callithrix jacchus). *Exp. Brain Res.* **122**, 351–61 (1998).
15. Zhong, G., Shevtsova, N. A., Rybak, I. A. & Harris-Warrick, R. M. Neuronal activity in the isolated mouse spinal cord during spontaneous deletions in fictive locomotion: insights into locomotor central pattern generator organization. *J. Physiol.* **590**, 4735–4759 (2012).
16. Danner, S. M. *et al.* Human spinal locomotor control is based on flexibly organized burst generators. *Brain* **138**, 577–588 (2015).
