# Supplementary material for: Rare phenomena of central rhythm and pattern generation in a case of complete spinal cord injury
Source: Nat Commun. 2023 Jun 6;14:3276. doi: 10.1038/s41467-023-39034-y (PMC10244420; doi:10.1038/s41467-023-39034-y)
Supplement: Supplementary file 3 — Reporting Summary [file 41467_2023_39034_MOESM3_ESM.pdf]

nature portfolio

Corresponding author(s): Ursula S. Hofstoetter
Last updated by author(s): Apr 24, 2023

Reporting Summary

Nature Portfolio wishes to improve the reproducibility of the work that we publish. This form provides structure for consistency and transparency in reporting. For further information on Nature Portfolio policies, see our Editorial Policies and the Editorial Policy Checklist.

Statistics

For all statistical analyses, confirm that the following items are present in the figure legend, table legend, main text, or Methods section.

n/a	Confirmed	
[ ]	[x]	The exact sample size (n) for each experimental group/condition, given as a discrete number and unit of measurement
[ ]	[x]	A statement on whether measurements were taken from distinct samples or whether the same sample was measured repeatedly
[ ]	[x]	The statistical test(s) used AND whether they are one- or two-sided<br>*Only common tests should be described solely by name; describe more complex techniques in the Methods section.*
[ ]	[x]	A description of all covariates tested
[ ]	[x]	A description of any assumptions or corrections, such as tests of normality and adjustment for multiple comparisons
[ ]	[x]	A full description of the statistical parameters including central tendency (e.g. means) or other basic estimates (e.g. regression coefficient) AND variation (e.g. standard deviation) or associated estimates of uncertainty (e.g. confidence intervals)
[ ]	[x]	For null hypothesis testing, the test statistic (e.g. F, t, r) with confidence intervals, effect sizes, degrees of freedom and P value noted<br>*Give P values as exact values whenever suitable.*
[x]	[ ]	For Bayesian analysis, information on the choice of priors and Markov chain Monte Carlo settings
[ ]	[x]	For hierarchical and complex designs, identification of the appropriate level for tests and full reporting of outcomes
[ ]	[x]	Estimates of effect sizes (e.g. Cohen's d, Pearson's r), indicating how they were calculated

Our web collection on statistics for biologists contains articles on many of the points above.

Software and code

Policy information about availability of computer code

Data collection	Electromyographic signals were amplified (Grass Instruments, Quincy, MA, USA) with a gain of 2000, filtered to a bandwidth of 30–700 Hz, and digitized at 2002 samples per second and channel using a Codas analog-to-digital converter system (Dataq Instruments, Akron, OH, USA).
Data analysis	IBM SPSS Statistics 27.0 for Windows (IBM Corporation, Armonk, NY, USA)<br>Matlab 2017b (The MathWorks, Inc., Natick, MA, USA)Grass Instruments, Quincy, MA, USA

For manuscripts utilizing custom algorithms or software that are central to the research but not yet described in published literature, software must be made available to editors and reviewers. We strongly encourage code deposition in a community repository (e.g. GitHub). See the Nature Portfolio guidelines for submitting code & software for further information.

Data

Policy information about availability of data

All manuscripts must include a data availability statement. This statement should provide the following information, where applicable:

- Accession codes, unique identifiers, or web links for publicly available datasets
- A description of any restrictions on data availability
- For clinical datasets or third party data, please ensure that the statement adheres to our policy

The authors declare that the data supporting the findings of this study are available within the paper and its supplementary information files. Source data are provided with this paper.

Human research participants

Policy information about studies involving human research participants and Sex and Gender in Research.

Reporting on sex and gender	single male individual
Population characteristics	spinal cord injury, hip pathology, rare form of spinal myoclonus, spinal cord stimulator, age: 23.4 years
Recruitment	retrospective analysis of clinical data
Ethics oversight	The study participant gave written informed consent for all interventions and procedures reported in the manuscript as well as for data processing and publication. Retrospective data analysis was approved by the Ethics Committee of the City of Vienna, Austria.

Note that full information on the approval of the study protocol must also be provided in the manuscript.

Field-specific reporting

Please select the one below that is the best fit for your research. If you are not sure, read the appropriate sections before making your selection.

[x] Life sciences [ ] Behavioural & social sciences [ ] Ecological, evolutionary & environmental sciences

For a reference copy of the document with all sections, see nature.com/documents/nr-reporting-summary-flat.pdf

Life sciences study design

All studies must disclose on these points even when the disclosure is negative.

Sample size	1. No statistical method was used to predetermine sample size. Data were selected because the participant presented with a very rare form of spinal myoclonus (prevalence < 1% in individuals with chronic spinal cord injury), expressed as self-sustained rhythmic activity in the lower limbs, and had an implanted epidural electrode lead.
Data exclusions	No data were excluded.
Replication	Nine neurological examinations over a period of three months showed reproducible outcomes.
Randomization	The clinical examinations used standardized protocols and were not randomized.
Blinding	The examiners were not blinded during experiments.

Reporting for specific materials, systems and methods

We require information from authors about some types of materials, experimental systems and methods used in many studies. Here, indicate whether each material, system or method listed is relevant to your study. If you are not sure if a list item applies to your research, read the appropriate section before selecting a response.

Materials & experimental systems

n/a	Involved in the study	
[x]	[ ]	Antibodies
[x]	[ ]	Eukaryotic cell lines
[x]	[ ]	Palaeontology and archaeology
[x]	[ ]	Animals and other organisms
[ ]	[x]	Clinical data
[x]	[ ]	Dual use research of concern

Methods

n/a	Involved in the study	
[x]	[ ]	ChIP-seq
[x]	[ ]	Flow cytometry
[x]	[ ]	MRI-based neuroimaging

Clinical data

Policy information about clinical studies

All manuscripts should comply with the ICMJEguidelines for publication of clinical research and a completedCONSORT checklist must be included with all submissions.

Clinical trial registration	This study was not conceived as a preospective clinical study and therefore was not registered.
Study protocol	Not a clinical study.
Data collection	Data were collected at the Neurological Center, Otto-Wagner-Hospital, Vienna, Austria, over a 3-month time period following standard clinical routine.
Outcomes	Rhythm and multi-muscle activation patterns of specific events within electromyographically recorded data
